# Sorafenib maintenance in FLT3-ITD mutated AML after allogeneic HCT: a real-world, single-center experience

**DOI:** 10.3389/fonc.2024.1391743

**Published:** 2024-06-24

**Authors:** Elisa Diral, Giulia Furnari, Alessandro Bruno, Raffaella Greco, Daniela Clerici, Sarah Marktel, Francesca Farina, Sara Mastaglio, Luca Vago, Simona Piemontese, Jacopo Peccatori, Consuelo Corti, Massimo Bernardi, Fabio Ciceri, Maria Teresa Lupo-Stanghellini

**Affiliations:** ^1^ Hematology and Bone Marrow Transplantation Unit, IRCCS San Raffaele Scientific Institute, Milan, Italy; ^2^ Faculty of Medicine and Surgery, Vita-Salute San Raffaele University, Milan, Italy

**Keywords:** sorafenib, maintenance, allogeneic stem cell transplantation, FLT3 ITD, acute myeloid leukemia

## Abstract

Despite allogeneic hematopoietic stem cell transplant (allo-HCT) and the development of novel FLT3 inhibitors in both induction (midostaurin) and in the relapsed/refractory setting (gilteritinib), FLT3-ITD mutated leukemia (FLT3-ITD+ AML) still represents a challenge for modern hematology. Sorafenib is, to this date, the only inhibitor that demonstrated efficacy in improving both progression-free and overall survival as post-HCT maintenance therapy, even if its use in this setting has not been approved so far by regulatory agencies. The aim of our study was to evaluate the feasibility, safety, and efficacy of sorafenib maintenance in preventing early relapse in FLT3-ITD+ AML after HCT in a single-center experience. We analyzed 26 consecutive patients who received post-HCT 2-year maintenance with sorafenib at our center between 2017 and 2023. The median time from HCT to sorafenib start was 130 days, and the median dosage was 200 mg per day. Two (8%) and three (12%) patients discontinued maintenance due to toxicity and disease relapse, respectively. Eight (31%) patients terminated the 2-year maintenance and stopped sorafenib, while 13 patients are still under treatment. Overall, 21/26 patients (81%) are alive and in stable complete remission as outlined by a 2-year disease-free survival of 83.61%. No major long-term toxicity was reported at the last follow-up. Our real-world experience supports the use of sorafenib as a feasible and effective therapeutic option in post-HCT maintenance for FLT3-ITD+ AML.

## Introduction

Acute myeloid leukemia (AML) with the Internal Tandem Duplication (ITD) mutation of the FMS-like tyrosine kinase 3 gene (FLT3-ITD) remains, to this day, one of the greatest challenges of modern hematology due to poor prognosis despite allogeneic hematopoietic stem cell transplantation (allo-HCT). FLT3 inhibitors have proven their efficacy in induction and salvage treatment, both in combination and in monotherapy. Three FLT3 inhibitors (FLT3-i) are currently approved by the FDA and EMA. Midostaurin and quizartinib have been approved by both the FDA and the EMA for induction therapy in combination with the “7 + 3” multi-chemotherapy regimen and for subsequent maintenance monotherapy based on the results of phase 3 trials RATIFY (1) and QuANTUM-First (2), respectively. Gilteritinib is a second-generation FLT3 inhibitor approved by the FDA in 2018 and by the EMA in 2019 as a monotherapy for patients with relapsed/refractory FLT3 mutated AML based on the results of the phase 3 ADMIRAL trial (3). However, none of these drugs have been approved as post allo-HCT maintenance. The use of sorafenib, a multi-targeted tyrosine kinase inhibitor, has been successfully explored in this setting in the phase 2 trial SORMAIN (4). Similarly a randomized, phase 3 trial showed long-lasting improved overall survival (OS) and leukemia-free survival (LFS—5-year follow up) in patients receiving sorafenib as post-HCT maintenance (5), confirming the results highlighted in a meta-analysis (6) evaluating 12 studies and more than 2,000 patients with a clear benefit on OS and LFS from post-transplant FLT3-i maintenance. Based on the increased relapse-free survival and good tolerability provided by sorafenib as post-HCT maintenance for FLT3 mutated AML, its use was recommended by the Acute Leukemia Working Party (ALWP)–European Society for Blood and Marrow Transplantation (EBMT) to optimize long-term disease control (7). It is worth noting that its use in maintenance after allo-HCT has not been approved so far by regulatory agencies. The aim of our study was to evaluate the feasibility, safety and efficacy of sorafenib maintenance in preventing early relapse in FLT3-ITD+ AML after allo-HCT in a single center experience.

## Methods

### Study design and participants

**Figure 1:**
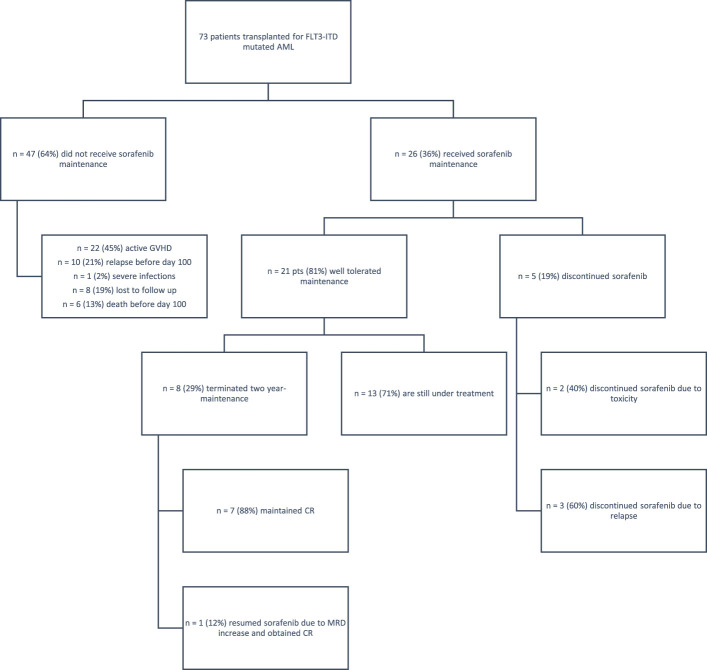
Eligibility and follow-up of patients. AML, acute myeloid leukemia; CR, complete remission; GvHD, graft-*versus*-host disease. A total of 79 patients with FLT3-ITD AML underwent transplantation; of these, 47 patients did not receive sorafenib due to various reasons— GvHD, early relapse, or severe infections; eight patients were lost to follow-up. The study group included 26 patients who received sorafenib; five patients discontinued maintenance for toxicity or relapse, while 21 patients tolerated the treatment well. Of these patients, 13 are still under treatment.

At our center, we performed allo-HCT in 297 AML patients between January 2017 and September 2023. Overall, 73 patients harbored a FLT-ITD mutation; of these, 47 patients (64%) could not receive maintenance due to various conditions (**Figure 1**), while 26 (36%) received sorafenib after allo-HCT. Considering this last cohort of patients, transplant was performed from all donor types, in particular, matched related, matched unrelated, haploidentical donor, and cord blood unit in 11 (42%), six (23%), six (23%), and three (12%) cases, respectively. According to center guidelines, a treosulfan-based conditioning regimen was adopted (8) in all cases, including all degrees of transplant conditioning intensities (9) (high *n* =15, 58%; intermediate *n* = 7, 27%; low *n* = 4, 15%). GvHD prophylaxis consisted of post-transplant cyclophosphamide (PtCy), sirolimus, and mycofenolate mofetil (MMF). Patients receiving matched related donors did not receive MMF, while patients receiving a cord blood unit did not receive PtCy. The inclusion criteria for sorafenib maintenance were as follows: (a) complete hematologic reconstitution (hemoglobin >10 g/dL, platelets >100,000/μL, and neutrophils >1,000/μL), (b) discontinuation of letermovir prophylaxis (day 100), (c) tapering of immunosuppressive therapy, and (d) absence of graft-*versus*-host disease (GvHD) requiring systemic treatment.

Disease status was re-evaluated at days +45 and +90 from transplant. In case of complete remission (CR), off label sorafenib was initiated at day +100 as recommended by the ALWP (5), after provision of an informed signed consent. Disease was evaluated at different timepoints (45 days from transplant, 3 and 6 months, 1 year, 1.5 years, 2 years, and then yearly until the 5th year). Patients with concomitant nucleophosmin1 (NPM1) mutation were monitored for the presence of the transcript by real-time quantitative polymerase chain reaction (RT-PCR) on peripheral blood and bone marrow at every disease re-evaluation time-point, and MRD negativity was defined as the ratio NPM1mut/ABL × 100 transcript <0.01%. For patients with unmutated NPM1 mutation, complete response was defined as morphological remission (marrow blasts <5%) associated with full donor chimerism.

### Statistical analysis

Overall survival (OS) was defined as the time from transplant to death from any cause or date of the last follow-up. The events for disease-free survival (DFS) were relapse or death. GRFS (GvHD/relapse-free survival) events were defined as the first event among grades III to IV acute GvHD, moderate to severe chronic GvHD, leukemia relapse, and death from any cause after allo-HCT. The OS, DFS, and GRFS rates were calculated using the Kaplan–Meier method and compared using log-rank test. A *p*-value lower than 0.05 was interpreted as significant.

Patient-, disease-, and transplant-related characteristics were compared using the *χ* ^2^ or Fisher’s exact test for categorical variables and the Mann–Whitney *U*-test for continuous variables.

## Results

Patient-, disease-, and transplant-related features are reported in **Table 1**. It is worth noting that, among the two groups of patients (patients treated with sorafenib and patients that did not receive sorafenib maintenance), no evidence of significant differences in terms of disease status at transplant, conditioning intensity, donor source, or post-transplant MRD positivity were observed.

**Table 1:** Patients’ features.

	Entire population 73 patients	Control group 47 patients	Sorafenib group 26 patients	*p*-value
**Sex (M/F)**	37/36 (50%/50%)	24/23 (51%/49%)	13/13 (50%/50%)	1
**Median age at HCT, years (range)**	52.5 (24–75)	53 (24–71)	51.5 (34–75)	0.73
**Mutational status at diagnosis**				0.34
NPM1+/FLT3+	39 (53%)	23 (49%)	16 (62%)	
FLT3+	34 (47%)	24 (51%)	10 (38%)	
**Disease status at transplant**				0.52
CR1	36 (50%)	22 (47%)	14 (54%)	
CR2	14 (19%)	10 (21%)	4 (15%)	
CR MRD+	5 (6%)	2 (4%)	3 (12%)	
Active disease	18 (25%)	13 (28%)	5 (19%)	
**TCI**				0.49
Low (1 to 2)	9 (12%)	5 (11%)	4 (15%)	
Intermediate (2.5–3.5)	26 (36%)	19 (40%)	7 (27%)	
High (4–6)	38 (52%)	23 (49%)	15 (58%)	
**GvHD prophylaxis**				0.69
PTCy based + rapamycin	66 (90%)	43 (91%)	23 (88%)	
Rapamycin + MMF	7 (10%)	4 (9%)	3 (12%)	
**Donor**				0.41
Matched related donor	14 (19%)	8 (17%)	6 (23%)	
Mismatched related donor	26 (36%)	20 (42%)	6 (23%)	
Matched unrelated donor	25 (34%)	14 (30%)	11 (42%)	
Cord blood unit	8 (11%)	5 (11%)	3 (12%)	
**NPM1-positive patients (*n*)**	39/73	23/47	16/26	
**MRD status post-HSCT**				1
Negative	21 (54%)	12 (52%)	9 (56%)	
Positive	18 (46%)	11 (48%)	7 (44%)	

HCT, hematopoietic stem cell transplantation; CR, complete remission (with MRD negativity for NPM1-mutated patients); MRD, minimal residual disease —for NPM1-mutated patients; TCI, transplant conditioning intensity; GvHD, graft-versus-host disease; MMF, mycophenolate mofetil.

Among patients in the sorafenib cohort, the median age at allo-HCT was 51 years (range, 34–75). The cohort included 13 male and 13 female patients. A total of 16 patients (62%) had concomitant NPM1 mutation, allowing for measurable residual disease (MRD) monitoring. Moreover, 21 patients were in CR at the time of transplantation (81%), three of which were with MRD positivity (11%) and five had active disease (19%). All patients were in complete morphological remission at the time of sorafenib initiation. Out of the NPM-positive patients, seven patients were MRD-positive at the time of sorafenib initiation. Interestingly, 21 patients had already received FLT3 inhibitors prior to allo-HCT, either in induction together with cytotoxic chemotherapy or at relapse: 18 patients had received prior midostaurin (69%), two had gilteritinib (8%) for relapsed/refractory disease, two had sorafenib (8%), and one patient had been included in a randomized trial with novel FLT3 inhibitors.

The median time from allo-HCT to start of sorafenib was 130 days (range, 49–1,026). Sorafenib was introduced at a minimum dosage of 200 mg every other day to reduce the drug–drug interaction with concomitant therapies (sirolimus, azoles, etc.), and it was progressively increased to 200 mg twice daily after immunosuppressive therapy discontinuation, with a median dosage of 200 mg daily. Two patients whose MRD was increasing were able to tolerate the maximum dosage of 400 mg twice daily without toxicities as well.

Sorafenib was overall well tolerated, with only two (8%) patients permanently discontinuing it for grade 3 – CTC AE toxicity (one gastro-intestinal and one cardiac toxicity). Six patients required a reduction to 200 mg every other day to mitigate the side effects, mainly gastro-intestinal discomfort. Other observed toxicities included hand–foot syndrome (*n* = 2, 8%; CTC AE G1), liver enzyme alteration (*n* = 2, 8%; CTC AE G2), and neutropenia (*n* = 1, 4%; CTC AE G3).

Eight patients completed the 2-year maintenance with sorafenib, and 13 patients are currently under treatment. At a median follow up of 34 months after sorafenib discontinuation, seven out of the eight patients that completed the 2-year maintenance (88%) maintained a continuous complete response. A single patient needed to restart sorafenib 96 days after maintenance completion due to early re-appearance of MRD positivity, which was confirmed at two subsequent controls. After resumption of sorafenib, MRD negativity was achieved soon after, and the patient is still alive and in CR, under sorafenib treatment. Overall, 21/26 patients (81%) with FLT3-ITD+ AML maintained stable CR, with no major long-term toxicity.

**Figure 2:**
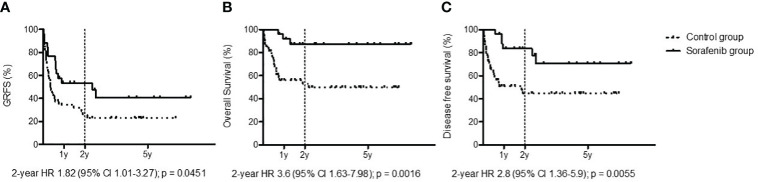
Outcomes: graft-*versus*-host disease/relapse-free survival **(A)**, overall survival **(B)**, and disease-free survival **(C)** among patients treated with sorafenib (black line) and patients untreated with sorafenib as post-allo-HCT maintenance (dotted line).

With regard to GvHD, 22 of the 73 patients transplanted for FLT3-ITD AML could not receive maintenance due to concomitant severe GvHD requiring high-dose steroids and drugs with possible interactions with sorafenib. In the cohort of patients receiving sorafenib, 8/26 patients (30%) previously had aGvHD, while 9/26 (35%) developed moderate to severe GvHD which did not require sorafenib discontinuation. At 2 years, GRFS was 53.57% in the cohort treated with sorafenib and 25.95% in the cohort that did not receive sorafenib [*p* = 0.0451, hazard ratio (HR): 1.82, 95% CI: 1.01–3.27] (**Figure 2A**).

Only three patients experienced disease progression during sorafenib therapy: the first one had positive MRD at the time of transplant, which increased steadily thereafter until overt hematological relapse. Due to a FLT3-ITD-positive relapse, an off-label treatment consisting of gilteritinib in combination with the bcl-2 inhibitor venetoclax was administered, but the patient died from disease progression. The second patient also had detectable MRD at the time of transplant, with transient MRD reduction after sorafenib initiation. Due to progressive MRD increase with initial FLT3-ITD positivity, the patient initiated gilteritinib therapy with consequent MRD clearance; the patient is still alive and in CR at 13 months from allo-HCT. The third patient had been included in a randomized clinical trial with novel FLT3-inhibitors; due to initial MRD positivity, without hematological relapse, the patient was switched to sorafenib maintenance and nevertheless later needed rescue with a second HCT.

With a median follow-up for the entire cohort (sorafenib group + control group) of 3 years, the sorafenib cohort reported a medium follow-up of 624 days (range, 139–2,499 days) and a median treatment exposure of 547 days (range, 3–1,686 days). We observed only one death due to disease progression. The 2-year OS for the cohort treated with sorafenib was 87.13%, while in the cohort that did not received sorafenib it was 52.82% (*p* = 0.0016, HR: 3.6, 95% CI: 1.63–7.98) (**Figure 2B**). The 2-year DFS for the cohort treated with sorafenib was 83.61%, while in the cohort that did not receive sorafenib it was 44.52% (*p* = 0.0055, HR: 2.8, 95% CI: 1.36–5.90) (**Figure 2C**).

## Discussion

Our real-world analysis confirms that sorafenib maintenance therapy is feasible, highlighting that the majority of candidate patients can start and complete the planned 2-year maintenance treatment. It is worth noting that, despite the limit of a retrospective study, the cohort of patients treated with sorafenib obtained a better outcome in terms of OS and DFS, which can be considered a direct measure of clinical benefit, outweighing (outlined as well by the significantly better GRFS) the possible toxicities exerted by sorafenib. Furthermore, the improvement of GRFS clarifies the conditional benefit of sorafenib capturing clinically meaningful events that impact the quantity and quality of survival after allo-HCT.

As reported by the two randomized trials (4, 5) and other groups (10, 11), sorafenib dosing can be individualized in the post-transplant setting according to patient tolerability. Sorafenib was well tolerated in our practice: drug-related toxicities and drug–drug interactions proved to be manageable through a customized approach, reducing the percentage of patients that permanently discontinued the maintenance. In fact, only 8% of patients discontinued sorafenib treatment in our study *versus* 22% of patients in the SORMAIN trial (4) and in contrast with other experiences (12).

Furthermore, preliminary results on the discontinuation of maintenance after 2 years of treatment confirm both the persistence of long-term remission and possibility to revert MRD positivity through resumption of the drug: sorafenib contributes to sustained long-lasting remissions of FLT3-ITD+ AML after allogeneic HCT.

Our real-world experience supports the use of sorafenib as a feasible and effective therapeutic option in post-HCT maintenance for FLT3-ITD+ AML across different donor sources.

## Data availability statement

The datasets generated for this study are available on request to the corresponding author. Requests to access these datasets should be directed to diral.elisa@hsr.it.

## Ethics statement

All patients were treated according to current Institutional programs upon written informed consent for transplant procedures, use of medical records and immunological studies for patients undergoing allogeneic HSCT within the non-interventional ALMON study, approved by San Raffaele Institutional Ethical Committee in date 19/10/2007. Sorafenib was given off-label after provision of an informed signed consent.

## Author contributions

ED: Conceptualization, Data curation, Investigation, Methodology, Supervision, Validation, Writing – original draft, Writing – review & editing. GF: Data curation, Validation, Writing – original draft. AB: Validation, Writing – review & editing. RG: Validation, Writing – review & editing. DC: Validation, Writing – review & editing, Formal analysis. SMar: Validation, Writing – review & editing. FF: Validation, Writing – review & editing. SMas: Validation, Writing – review & editing. LV: Validation, Writing – review & editing. SP: Validation, Writing – review & editing. JP: Validation, Writing – review & editing. CC: Validation, Writing – review & editing. MB: Validation, Writing – review & editing. FC: Validation, Writing – review & editing. ML-S: Conceptualization, Formal analysis, Investigation, Methodology, Supervision, Validation, Visualization, Writing – original draft, Writing – review & editing.
